# Luteolin ameliorates lipopolysaccharide-induced microcirculatory disturbance through inhibiting leukocyte adhesion in rat mesenteric venules

**DOI:** 10.1186/s12906-020-03196-9

**Published:** 2021-01-14

**Authors:** Jie Su, Han-Ting Xu, Jing-Jing Yu, Mei-Qiu Yan, Ting Wang, Ya-Jun Wu, Bo Li, Wen-Jie Lu, Chuan Wang, Shan-Shan Lei, Si-Min Chen, Su-Hong Chen, Gui-Yuan Lv

**Affiliations:** 1 College of Pharmaceutical Sciences, Zhejiang Chinese Medical University, Hangzhou, 310053 People’s Republic of China; 2 Suzhou Wuzhong People’s Hospital, Suzhou, 215128 China; 3 Collaborative Innovation Center of Yangtze River Delta Region Green Pharmaceuticals, Zhejiang University of Technology, Hangzhou, 310014 Zhejiang China

**Keywords:** Luteolin, LPS, Leukocyte adhesion, Microcirculatory disturbance

## Abstract

**Background:**

Microcirculatory disturbance is closely associated with multiple diseases such as ischemic and septic stroke. Luteolin (3,4,5,7-tetrahydroxyflavone) is a vascular protective flavonoid present in several dietary foods. However, how luteolin plays a role in microcirculatory disturbance is still unknown. The purpose of this study was to find out the influence of luteolin on the lipopolysaccharide (LPS)-induced microcirculatory disturbance, focusing on its effect on leukocyte adhesion and the underlying mechanism of this effect.

**Methods:**

After injecting LPS into rats, we used an inverted intravital microscope to observe the velocity of red blood cells in venules, numbers of leukocytes adherent to and emigrated across the venular wall, hydrogen peroxide production in venular walls and mast cell degranulation. Intestinal microcirculation blood flow was measured by High-resolution Laser Doppler Perfusion Imaging. Histological changes of small intestine and mesenteric arteries were evaluated. Additionally, cell adhesion stimulated by LPS was tested on EA.hy926 and THP-1 cells. The production of pro-inflammatory cytokines, adhesion molecules and the activation of TLR4/Myd88/NF-κB signaling pathway were determined.

**Results:**

The results showed luteolin significantly inhibited LPS-induced leukocyte adhesion, hydrogen peroxide production and mast cell degranulation, and increased intestinal microcirculation blood flow and ameliorated pathological changes in the mesenteric artery and the small intestine. Furthermore, luteolin inhibited the release of pro-inflammatory cytokines, the expression of TLR4, Myd88, ICAM-1, and VCAM-1, the phosphorylation of IκB-α and NF-κB/p65 in LPS stimulated EA.hy926.

**Conclusions:**

Our findings revealed that it is likely that luteolin can ameliorate microcirculatory disturbance. The inhibitory effects of luteolin on the leukocyte adhesion stimulated by LPS, which participates in the development of microcirculatory disturbance, are mediated through the regulation of the TLR4/Myd88/NF-κB signaling pathway.

**Supplementary Information:**

The online version contains supplementary material available at 10.1186/s12906-020-03196-9.

## Background

Microcirculation is composed of arterioles, capillaries, and venules, functioning essentially as oxygen and source supply depending on the unique physiological need of the supplied organs [1]. It is composed of endothelial cells (ECs), basement membranes, pericytes, and smooth muscle cells. Microcirculatory disturbance, which can cause multiple organ damage, is a complex pathological process involving abnormal leukocyte-EC interactions, excessive production of peroxide, degranulated mast cells, and hyperpermeability of microvessel wall [2, 3]. It is also worth noting that leukocyte-EC interactions, especially leukocyte adhesion is a crucial step in microcirculatory disturbance processes.

The intact structure and optimal function of ECs ensure the normal microcirculation. The morphological characteristics of endothelial cells [4] and the expression of adhesion molecules determine that the leukocyte-EC interaction occurs more in venules [5, 6] than in arterioles [7]. The raised in leukocyte-EC interaction in inflamed vessels is identified as risk factors for thrombosis [8, 9]. Therefore, as a response to inflammatory stimulation, the dysfunction of venular EC may lead to thrombosis, stroke and other related diseases. In previous studies, venules have been found to be closely related to some diseases [10] such as ischemic stroke [11]. In the process of treating related diseases, improving the disturbance of microcirculation is an important measure to achieve a good prognosis.

Injection of Lipopolysaccharide (LPS) on animals is widely used as a model in the field of microcirculation disturbance research. LPS, located at the cell wall of gram-negative bacteria [9], can cause systemic inflammatory and septic shock accompanied with microcirculatory disturbance [12]. LPS could promote the expression of adhesion molecules [13], resulting in adhesion of leukocytes to ECs [14, 15]. Peroxides and proteases are produced when leukocytes adhere to the venular wall, which damages ECs and aggravate microvessel injury [16]. Moreover, these could also cause mast cells to degranulate and release pro-inflammatory cytokines [17]. These factors subsequently cause microcirculatory disturbance. Accordingly, suppression of adhesion of leukocytes and ECs is of great importance to improve LPS-induced microcirculatory disturbance.

Luteolin (3,4,5,7-tetrahydroxyflavone) is the main flavonoid contained in wild chrysanthemum and peanut shells [18, 19]. Moreover, it is one of the most common flavonoids in many dietetic foods, such as broccoli, olive oil, celery, parsley, green pepper, various herbs, dandelion, and Japanese honeysuckle [20]. It exhibits a variety of biological and pharmacological activities, including anti-inflammatory [21, 22], antioxidant, antihypertension [23], cardiovascular protection [24], anticancer and other activities. The improvement of vascular endothelial inflammation is widely considered to be the most significant advantage of luteolin. Vogl and coworkers reported that there was a decreasing expression of interleukin-8 (IL-8) and E-selectin protein caused by luteolin in ECs after LPS stimulation [25]. Luteolin could also protect against tumor necrosis factor-alpha (TNF-α)-induced vascular inflammation via interfering with NF-κB-mediated pathway [26].

In our preliminary experiment, we found that luteolin protects blood vessels by suppressing hypertensive vascular remodeling [23]. However, the role of luteolin in microcirculatory disturbance is still not clear and its underlying mechanism remains unexplored. Therefore, in this study, we employed a Sprague-Dawley (SD) rat inflammatory model induced by LPS to evaluate whether luteolin is capable of improving microcirculatory disturbance in rat mesenteric venules in vivo. Besides, we tried to elucidate its molecular mechanism of action with the LPS-induced EA.hy926 model in vitro. This research aimed to illuminate the mechanism of luteolin against microcirculatory disturbance and provide a theoretical basis for further development and utilization of luteolin.

## Methods

### Drug and chemicals preparation

Luteolin (LUT, purity>98%, L9283) and LPS were obtained from Sigma Chemical Co (St.Louis, MO, USA). We used DMSO to dissolve the compounds in vitro as the stock solution, which was stored at − 20 °C and diluted before use.

Luteolin enriched extracts (TLUT, purity>50%) were extracted from peanut shells according to the method described by Su et al. [23]. The peanut shells were purchased from New Nongdu Co., Ltd. (Hang Zhou, China).

Dihydrorhodamine 123 (DHR) was purchased from Molecular Probes Ltd. (Eugene, OR, USA). LPS (O55:B5), and toluidine blue were from Sigma Chemical Co. (St Louis, MO, USA).

### Animals

Male Sprague-Dawley (SD) rats, with body weights ranging from 160 to 180 g, were provided by Experimental Animal Center of Zhejiang Province (Certificate no. SCXK 2014–0001, Hangzhou, China). They were acclimatized for 2 weeks before experiments. During this period, all of the rats were fed with standard diet and water. Animals were housed in a room with constant temperature, humidity, and light/dark cycle. All animal care and experimental procedures were approved by the Institutional Animal Care and Use Committee (IACUC) in the Zhejiang Chinese Medical University (permission number: ZSLL-2015-84).

### Animal treatments and experimental design

Forty SD rats were randomly divided into four groups by SPSS statistics (version 17.0) software’s random number generator, and the experimental data analyses were conducted in a blinded manner. Group one (G1) was set as the control group. Rats injected with LPS and gavaged with distilled water, were designated as the LPS group (G2). Group three (G3) and group four (G4) were orally administered with TLUT (at the doses of 75 and 45 mg/kg, respectively) for 20 days in addition to LPS treatment.

The operation was similar to what Kai Sun performed [27]. Briefly, rats were anesthetized with an intraperitoneal injection of pentobarbital sodium (50 mg/kg) and then injected by tail vein with LPS (1 mg/kg) dissolved in saline while the rats of the control group were injected with saline alone. After that, the abdomen was opened by a midline blade (2–3 cm in length), and the ileocecal portion of the mesentery was mounted with care on a transparent plastic stage designed for the rat. The mesentery was kept warm in a constant temperature device and humidified by continuous dropping with saline at 37 °C. Microcirculatory hemodynamics in the mesentery was perceived by using a dynamic visualization of the microcirculation tracking system (Gene&I, Beijing, China). The single unbranched venules with 30 to 50 μm wide and about 200 μm long were selected for observation.

### Intravital observation of mesenteric microcirculatory

The microcirculatory hemodynamics in rat mesentery was inspected and recorded continuously [28]. During the playback video images, the venular diameters at 20 min interval after 10 min of LPS injection was measured using Image-Pro Plus 6.0 software. The average diameter from three sites was calculated.

The movement of red blood cells (RBCs) was recorded (500 frames/sec) by a high-speed video camera system (Integrated Design Tools, Inc., USA). The velocity of RBC was analyzed by Image-Pro Plus 6.0 software. The average value was calculated from three different RBCs in the same time frame.

With the recorded videotaped images, we defined the rolling leukocytes as those that could be seen moving 10 s within the same segment of 200 μm long vessel. In addition, the adherent leukocytes were defined as those adhered to the same site for more than 10 s, and an average of 3 times of rolling and adherent leukocytes were counted.

To quantify the hydrogen peroxide production in venular wall, 10 μM DHR, a H_2_O_2_-sensitive fluorescent probe, was dropped to the mesenteric surface after dynamic observing 90 min. DHR fluorescence intensity on the venular wall (D_B_) and extra-vascular (D_W_) was measured using an image processor. D_B_-D_W_ reflected the vascular wall superoxide production.

After DHR fluorescence examination, the mast cells in the mesentery were stained with 0.1% toluidine blue. Then we assessed the mast cell degranulation by averaging ratios of the number of degranulated mast cells to the total number of mast cells from the six captured pictures.

### High-resolution laser doppler perfusion imaging

Intestinal microcirculation blood flow was evaluated by laser Doppler with a moorFLPI V3 software (Moor Instruments Ltd., UK) [29, 30]. After intravital observation of mesenteric microcirculation, the mesentery of rats was chosen to examine with an appropriate distance between the scanner and the mesentery of rats. The intensity of blood flow is presented with the different colors expanding from blue to green (low blood flow) and yellow to red (highest blood flow). Intestinal average microcirculation blood flow within each 15 s from three regions was determined by mFLPIReviewV3 image analysis software.

### Histological evaluation

The rats were euthanized after completing the experiment, anesthetized with an intraperitoneal injection of pentobarbital sodium (150 mg/kg), approved methods for euthanasia by the Animal Care and Welfare Committee in the Zhejiang Chinese Medical University. The small intestine and mesenteric artery were excised and fixed in 10% formalin. After fixing, tissues were embedded in paraffin and cut into 4 μm thick sections. Which were stained with hematoxylin and eosin (Nanjing Jiangcheng Bioengineering Institute, Nanjing, China). Stained sections were visualized under the microscope (100X and 400X).

### Cell culture

Human umbilical vein cell fusion cell (EA.hy926) with vascular endothelial cell characteristics (purchased from the Type Culture Collection of the Chinese Academy of Sciences, Shanghai, China) were maintained in Dulbecco’s modified Eagle’s medium (DMEM; Life Technologies Corporation, Beijing, China.), Human monocytic leukemia cell (THP-1; Type Culture Collection of the Chinese Academy of Sciences, Shanghai, China) were cultured in RPMI 1640 Medium (Gino Biological Pharmaceutical Co., Ltd. Hangzhou, China). Both mediums were supplemented with 10% fetal bovine serum (FBS) (Gemini Bio-Products, Australia.), 100 U/mL penicillin, 100 μg/mL streptomycin Cells were maintained in a 37 °C and 5% CO_2_ incubator.

### Cytotoxicity assay

In the experiment, we used MTT to assess the viability of EA.hy926. EA.hy926cells were passaged and then inoculated into 96-well plates at a concentration of 1× 10^4^/well 24 h after inoculation, cells were treated with different concentrations of LUT (0.1, 1, 5, 10, 20, 30 μM) in serum-free medium for another 24 h. The viability of cells was measured as described previously [23].

### Cell adhesion assay

In order to evaluate the effect of luteolin on adhesive ability, we used LPS to stimulate EA.hy926 and THP-1 cells. Briefly, EA.hy926 cells were seeded at 1.5 × 10^4^ /well in 96-well plates for 12 h. They were pre-treated with 5, 10, 20 μM LUT for 12 h, then co-incubated with 3 μg/mL LPS for 6 h. Meanwhile, THP-1 labeled with 5 μg/mL DAPI overnight was added into EA.hy926 plate (5 × 10^4^ /well) and incubated for 30 min. Cells were carefully washed 3 times with PBS to remove non-adherent ones. Under the inverted fluorescence microscope (Olympus Corporation, Japan), nine random views were captured in each well at 200 × magnification and images were analyzed with Image-Pro Plus 5.1 software.

### Enzyme-linked immunosorbent assay (ELISA)

EA.hy926 (1.5 × 10^4^ each well) were grown in 96-well plates for 12 h and incubated with LUT at 5, 10, 20 μM and 3 μg/ml LPS for 24 h. Then cell supernatants were collected. TNF-α and IL-8 level were determined using ELISA kits (Shanghai Yuanye Biological pharmaceutical technology co., Ltd., Shanghai, China) according to the manufacturer’s protocol, respectively.

### Cell protein extraction and western blotting analysis

The method resembled that of Jie Su [23]. In short, cells treated with various concentrations of LUT were collected. Cell proteins were extracted with RAPI buffer then separated by SDS-PAGE. Proteins were electro-transferred onto a polyvinylidene-difluoride membrane. After blocking with 5% BSA, the membranes were cultivated overnight at 4 °C with corresponding first antibodies against the NF-κBp65, p-NF-κBp65, Myd88, IκB-α, p-IκB-α (Cell Signaling Technology, Canada), toll-like receptor 4 (TLR4), intercellular adhesion molecule 1 (ICAM-1), vascular cell adhesion molecule 1 (VCAM-1) (Santa Cruz Biotechnology, USA). β-actin (Abcam, UK) was used as a loading control. Appropriate secondary antibody labeled with IRDye® 800CW (Li-cor, USA) were incubated with the membranes after TBST washes. Lastly, the protein bands were examined by LI-COR Odyssey CLx Infrared Imaging System.

### Statistical analysis

All quantitative results were presented mean ± standard deviation. The differences between groups were analyzed by One-way analysis of variance (ANOVA) with LSD *t*-test or Dunnet *t*-test by SPSS 17.0 software. When homogeneity of variance assumptions is satisfied, the LSD *t*-test will be used; otherwise, when heterogeneity of variance assumptions is satisfied, the Dunnet *t*-test will be applied. The value of *P*< 0.05 was deemed to be statistically significant. Diagrams were generateded by Graph Prims.

## Results

### Effect of luteolin on the leukocyte adhesion to venular wall

**Fig. 1:**
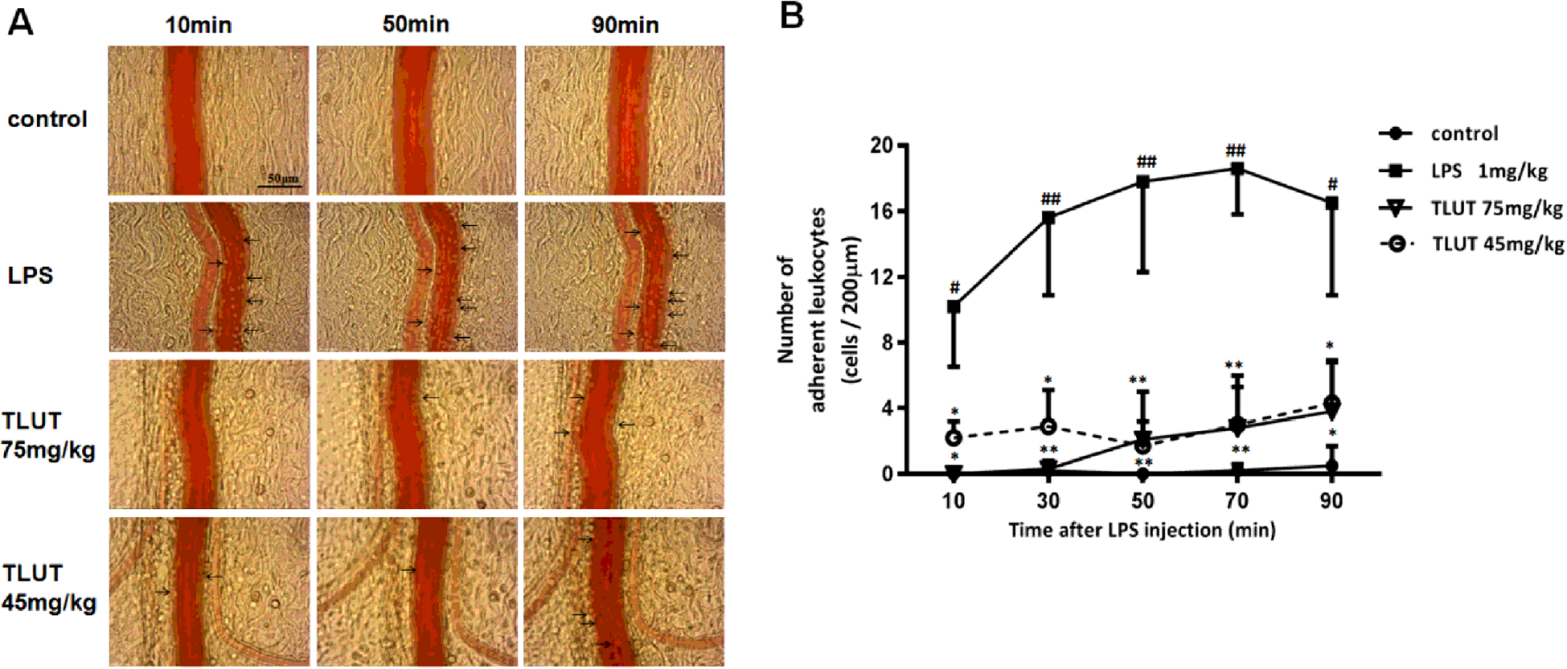
The effect of luteolin on LPS-induced leukocyte adhesion to the rat mesenteric venular wall. **a** Representative images illustrating the effect of luteolin on leukocyte adhesion to the venular wall induced by LPS in rat mesentery at 10, 50, 90 min after LPS injection, respectively (400X). Arrows: leukocyte adhesion to venular wall. **b** Time course of the number of leukocytes adhesion to the rat mesenteric venular wall. The data were expressed as means ± SEM (*n* = 6). Significant differences were indicated by ^#^*P*< 0.05, ^##^*P*< 0.01 as compared with control group, ^*^P< 0.05, ^**^P< 0.01 as compared with LPS group

The effect of luteolin on the adhesion between leukocytes and ECs was investigated both in vivo and in vitro. In vivo study, pictures of leukocyte adhesion to venular wall of each group at 10, 50, 90 min after LPS injection were presented in Fig.1a. Under various conditions, we did not observe adherent leukocytes in control group. In contrast, after LPS injection, numerous adherent leukocytes were observed in LPS group, while only a few adherent leukocytes in TLUT groups. Moreover, no adherent leukocyte was observed at 10 min after LPS injection in TLUT 75 mg/kg group, while still some adherent leukocytes remained at 10 min in TLUT 45 mg/kg group.

Furthermore, Fig.1b depicted the time course of leukocytes adhesion to the venular wall under different conditions, confirming the above qualitative evaluation. There were few adherent leukocytes in the control group, if any, throughout the whole observation period. Conversely, the number of adherent leukocytes is much higher in LPS group at each time point compared with control group (*P*< 0.01 or *P*< 0.05), and it reached the highest level of 18.6 ± 2.8 cells / 200 μm at 70 min after LPS injection. After LPS stimulation, the number of adherent leukocytes increased significantly from 10 min to 90 min. However, it declined significantly in groups treated with 75 mg/kg and 45 mg/kg TLUT (*P*< 0.01 or *P*< 0.05). Furthermore, the decrease was more dramatic in TLUT 75 mg/kg group than TLUT 45 mg/kg group. Impressively, treatment with TLUT resulted in marked inhibition of the LPS-induced leukocyte adhesion in vivo.

**Fig. 2:**
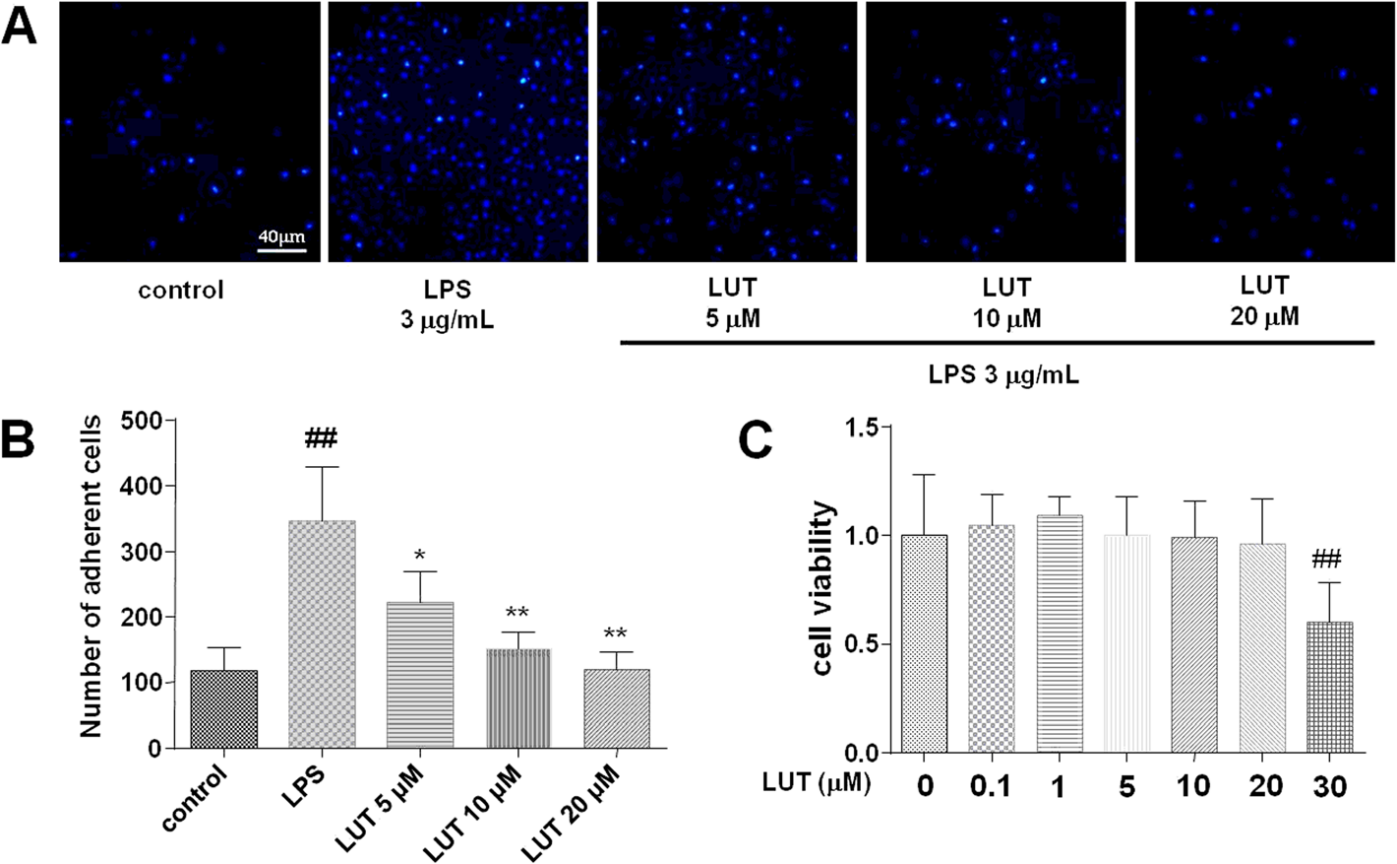
The effect of luteolin on LPS-induced cell adhesion between EA.hy926 and THP-1. **a** Representative images of cell adhesion between EA.hy926 and THP-1. Pictures were captured at 200x magnification. **b** A quantitative evaluation of adherent cells. The data were expressed as means ± SEM (*n* = 9). Significant differences were indicated by ^##^*P*< 0.01 as compared with the control group, ^*^P< 0.05, ^**^P< 0.01 as compared with the LPS group. **c** The effect of luteolin on cell viability in EA.hy926. The data were expressed as means ± SEM (*n* = 12). Significant differences were indicated by ^##^*P*< 0.01 as compared with the control group

To prove the effect of luteolin on cell adhesion in vitro, we examined the cell adhesion in EA.hy926 and THP-1 cells. First of all, we detected the cytotoxicity of luteolin on EA.hy926 cells. As presented in Fig.2c, Compared with control group, it had a significant inhibition of the cell viability of EA.hy926 after treatment with LUT at 30 μM (*P*< 0.01). However, there was no marked difference between other groups and the control group in cell viability. The results turned out that cytotoxicity of luteolin was at a concentration of up to 30 μM. Therefore, LUT at 5 μM, 10 μM, and 20 μM were used in subsequent experiments. As shown in Fig.2b, EA.hy926 stimulated by 3 μg/mL LPS attracted more THP-1 cells in comparison with the control group (*P*< 0.01). The numbers of LPS-induced adherent cells significantly decreased when EA.hy926 cells were incubated 24 h with 5 μM, 10 μM, and 20 μM LUT (*P*< 0.01 or *P*< 0.05).

In conclusion, luteolin could significantly inhibit leukocyte adhesion to the venular wall in vivo and in vitro induced by LPS, demonstrate a direct inhibitory effect on leukocytes adhesion to the endothelium.

### Effect of luteolin on the leukocyte rolling along venular wall

As illustrated in Table 1, the number of rolling leukocytes along a 200 μm venular wall in the control group was less than five per 10s. Compared with the control group, the number of rolling leukocytes increased in the LPS group which became most significant at 10 min and 30 min after LPS injection, but there was no significant difference in statistics. Moreover, the data showed that there was no significant statistical difference between the TLUT group and the LPS group at any time point, but it was found that 10 and 30 min after the injection of LPS, the treatment with TLUT could alleviate the rolling leukocyte increase induced by LPS to some extent, but this effect was not conspicuous.

**Table 1:** The effect of luteolin on leukocyte rolling along venular wall

Groups	Time after LPS injection (min)				
	10	30	50	70	90
Control	1.9±1.2	1.7±1.1	1.8±1.0	1.6±1.1	2.4±1.9
LPS 1 mg/kg	9.1±4.4	5.2±2.1	3.8±3.5	2.4±1.8	2.2±2.0
TLUT 75 mg/kg	5.4±4.2	4.2±2.4	5.0±4.1	3.8±2.0	5.9±3.3
TLUT 45 mg/kg	4.4±3.2	3.7±2.1	4.1±2.0	3.7±2.2	4.7±1.9

The data were expressed as means ± SEM (*n* = 6)

### Effect of luteolin on the hydrogen peroxide production in the venular wall

**Fig. 3:**
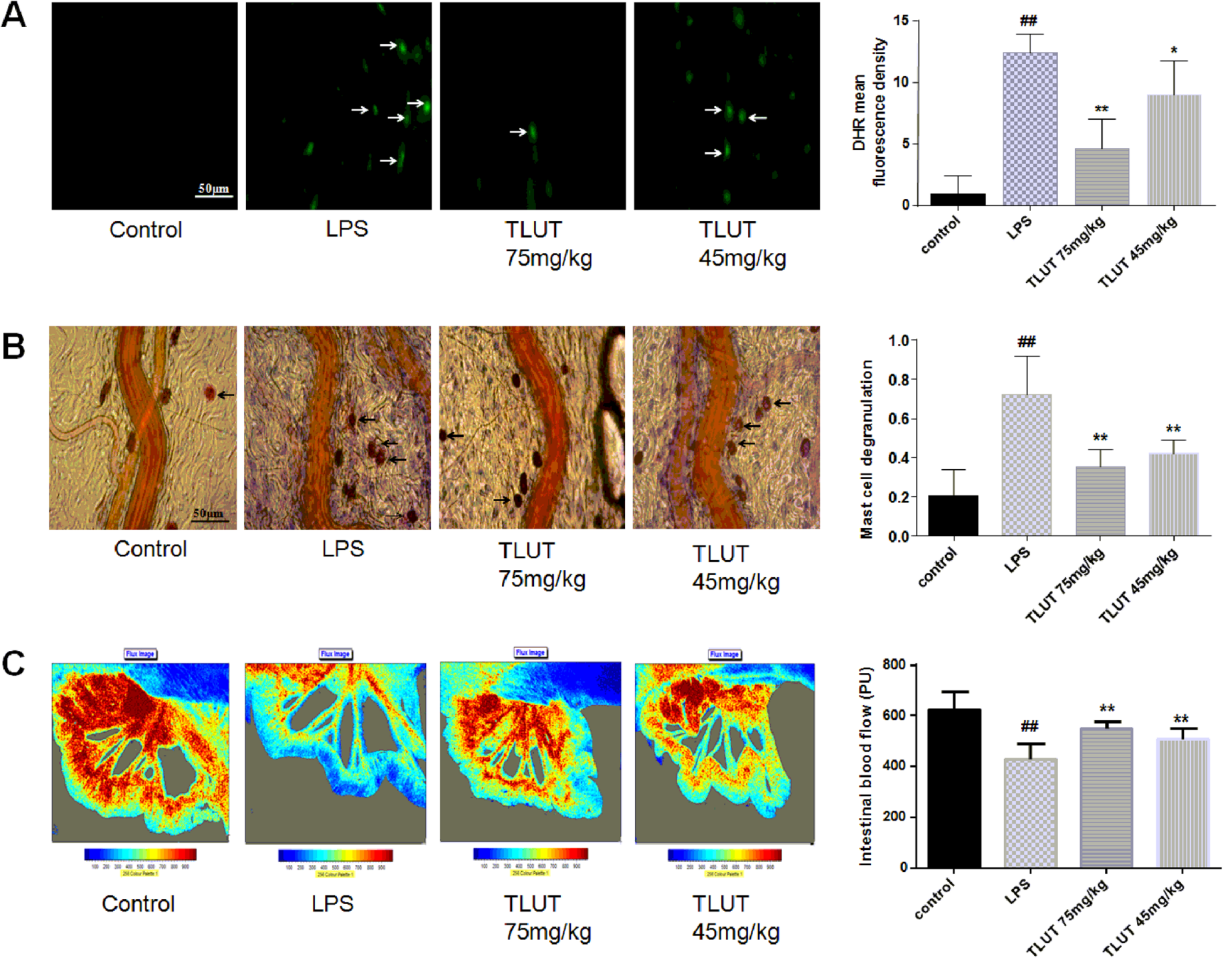
The effect of luteolin on LPS-induced hydrogen peroxide production, mast cell degranulation and intestinal microcirculation blood flow. **a** The effect of luteolin on LPS-induced hydrogen peroxide production. Pictures were captured at 400x magnification. Arrows: DHR fluorescence on the venular walls. **b** The effect of luteolin on LPS-induced mast cell degranulation. Pictures were captured at 400x magnification. Arrows: degranulated mast cell. **c** The effect of luteolin on LPS-stimulated intestinal microcirculation blood flow. All of the representative images of each group were presented with the respective quantification showing right correspondingly. All of the data were expressed as means ± SEM (*n* = 6). Significant differences were indicated by ^##^*P*< 0.01 as compared with control group, ^*^P< 0.05, ^**^P< 0.01 as compared with LPS group

As presented in Fig.3a, almost no detectable DHR fluorescence in rat mesenteric venular wall was observed in the control group. In contrast, the DHR fluorescence intensity increased impressively in the LPS group after 90 min of LPS injection (*P*< 0.01) and was high to 12.4 ± 1.5. However, treatment with 75 mg/kg and 45 mg/kg TLUT markedly inhibited LPS-induced hydrogen peroxide production (*P*< 0.01 or *P*< 0.05), and the DHR fluorescence intensity in TLUT 75 mg/kg group was low to 4.6 ± 2.4.

### Effect of luteolin on the mast cell degranulation

Mast cell degranulation after 90 min of LPS injection was analyzed and expressed as the ratio of the number of degranulated mast cells to the total number of mast cells, as shown in Fig.3b. In the control group, degranulated mast cells were very small. After LPS injection, the percentage of degranulated mast cells significantly increased (*P*< 0.01), which was high to 71.9% ± 19.8%. However, treatment with 75 mg/kg and 45 mg/kg TLUT markedly suppressed the LPS-induced degranulation of mast cells (*P*< 0.01). The value was 35.0% ± 8.9 and 42.2% ± 6.6%, respectively.

### Effect of luteolin on the intestinal microcirculation blood flow and RBC velocity

We measured the intestinal microcirculation blood flow by the laser Doppler perfusion imaging. Figure 3c illustrates the intestinal microcirculation blood flow, which was examined after observing mast cell degranulation. The control SD rats had the adequate blood flow. On the contrary, the blood flow in the LPS group impressively declined (*P*< 0.01), which was low to 431.1 ± 62.2 PU. However, the blood flow was apparently increased with the treatment of 75 mg/kg and 45 mg/kg TLUT (*P*< 0.01). The value was 554.2 ± 27.2 PU and 511.8 ± 42.9 PU, respectively.

As is known, the blood flow velocity is a direct factor affecting blood flow. Thus we further examined the RBC velocity in venules by a high-speed video camera system. Table 2 demonstrated the period in which RBC velocity changes in mesenteric venules. The velocity of RBC in LPS group progressively declined at 10 min and 30 min after LPS injection in comparison with the control group (*P*< 0.01 or *P*< 0.05). However, This LPS-induced decrease in RBC velocity was without significant attenuated by treatment with TLUT. In addition, saline injection led to a progressive decrease in RBC velocity, and a remarkable difference was observed 50 min, 70 min and 90 min after saline injection compared with 10 min (*P*< 0.01 or *P*< 0.05).

**Table 2:** The effect of luteolin on RBC velocity in mesenteric venules of rats injected with LPS (mm/s)

Groups	Time after LPS injection (min)				
	10	30	50	70	90
Control	2.96±0.60	2.57±0.44	2.02±0.47^$$^	2.15±0.58^$^	1.98±0.56^$$^
LPS 1 mg/kg	1.35±0.34^##^	1.41±0.31^#^	1.39±0.26	1.37±0.31	1.25±0.28
TLUT 75 mg/kg	1.65±0.66	1.70±0.96	1.82±0.89	1.72±0.88	1.55±0.96
TLUT 45 mg/kg	1.38±0.77	1.41±0.85	1.10±0.34	1.04±0.47	1.07±0.34

The data were expressed as means ± SEM (n = 6); ^#^*P*< 0.05 vs. control group;^##^*P*< 0.01 vs. control group;^$^*P*< 0.05 vs. 10 min;^$$^*P*< 0.01 vs. 10 min

The data showed that luteolin could improve intestinal microcirculation blood flow suppressed by LPS, but it had no significant effect on RBC velocity, suggesting that the effect of luteolin on improving intestinal microcirculation blood flow is not related to RBC velocity.

### Effect of luteolin on the histology of mesenteric artery and small intestine

**Fig. 4:**
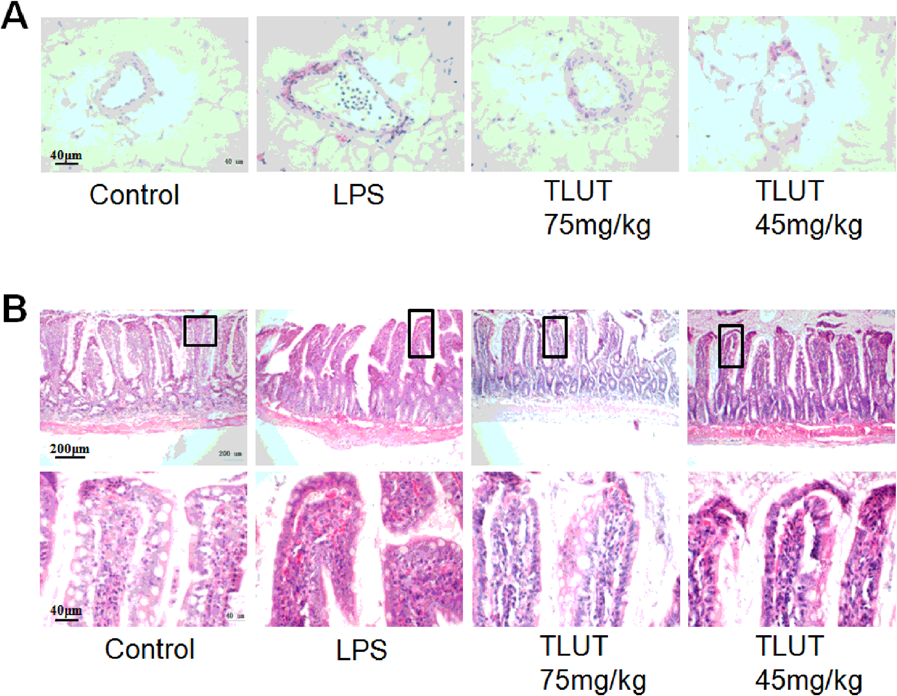
The effect of luteolin on LPS-stimulated mesenteric artery and small intestine pathological changes. **a** Representative images of HE staining of the mesenteric artery in each group (400X). **b** Typical images of HE staining of the small intestine in each group (100X). The area in the rectangle of each image was enlarged and shown below correspondingly (400X)

The HE staining for rat mesenteric artery and small intestine were presented in Fig.4. As shown in Fig.4a, in the LPS group, membrane cells of the mesenteric artery had the irregular arrangement, and inflammatory cells infiltrated compared with the control group. Moreover, Fig.4b illustrates the situation of the disintegration of intestinal villi existed in the LPS group. However, it is comfortable that the situation was ameliorated by 75 mg/kg and 45 mg/kg TLUT. Especially, the effect in 75 mg/kg TLUT group was more significant.

### Effect of luteolin on the LPS/TLR4/NF-κB signaling pathway in EA.hy926

**Fig. 5:**
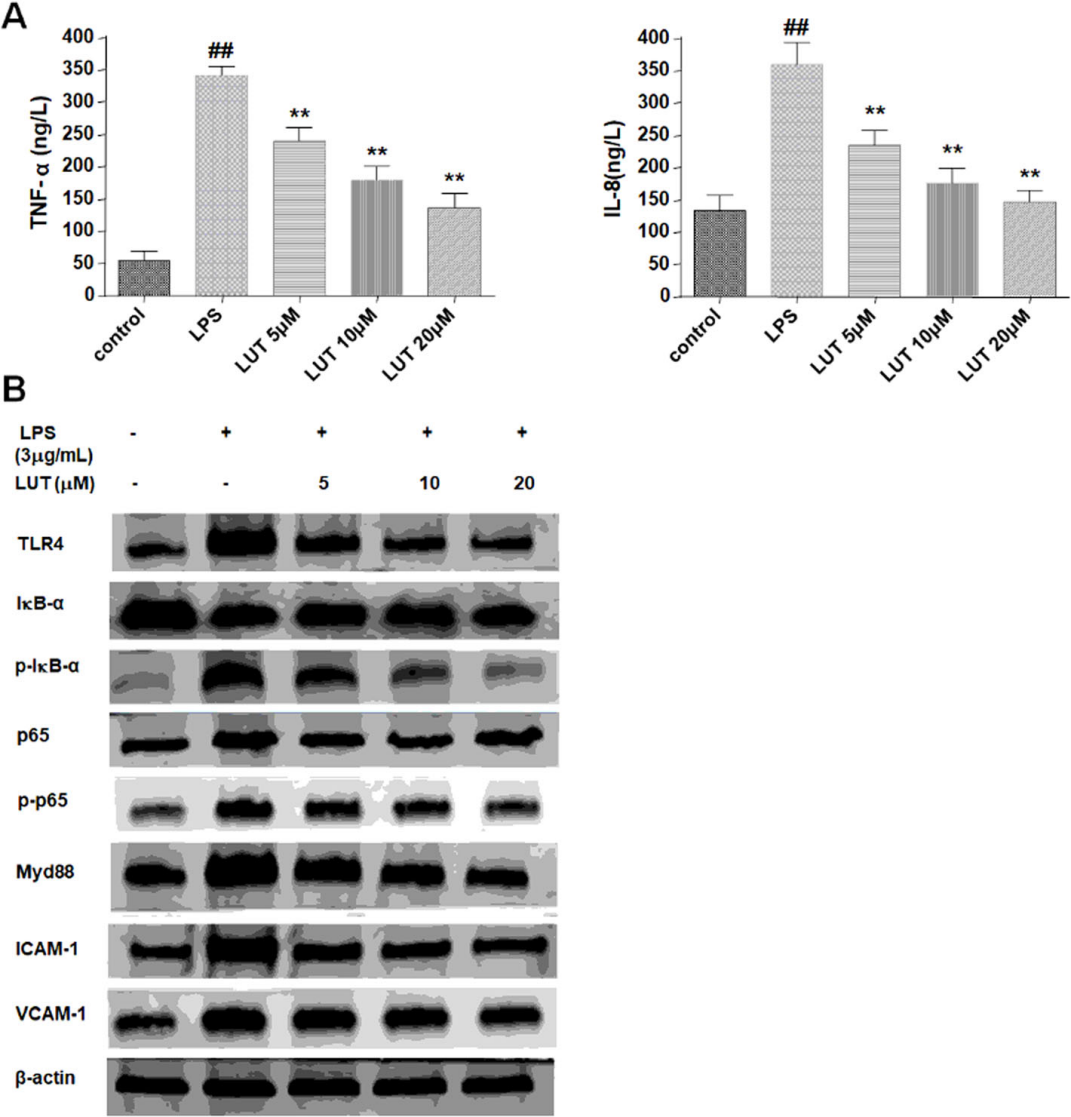
The effect of luteolin on LPS/TLR4/NF-κB pathway. **a** The TNF-α and IL-8 level in cultural supernatants of EA.hy926 cells. The data were expressed as means ± SEM (*n* = 8). Significant differences were indicated by ^##^*P*< 0.01 as compared with control group, ^**^P< 0.01 as compared with LPS group. **b** The expression of TLR4, Myd88, IκB-α, NF-κB/p65, ICAM-1 and VCAM-1, and activation of IκB-α and NF-κB/p65 in EA.hy926 cells

TLR4 participated in the signaling transduction induced by LPS, which led to the activation of NF-κB. NF-κB, a ubiquitous transcription factor, controls the expression of a large number of adhesion molecules and pro-inflammatory cytokines. In order to explore the anti-adhesion mechanisms of luteolin, we examined the expression of related factors of LPS/TLR4/NF-κB signaling pathway in EA.hy926 stimulated with LPS by western blot, including TLR4, Myd88, ICAM-1, VCAM-1 and activation of IκB-α and NF-κB/p65. The results of western blot were illustrated in Fig. 5. The expression of TLR4, Myd88, ICAM-1 and VCAM-1 were increased in LPS stimulated EA.hy926 cells. While treatment of LUT markedly reduced their expression, especially in the high concentration of LUT at 20 μM.

Phosphorylation of IκB-α through proteolytic degradation leads to the nuclear translocation of NF-κB. Exposure to LPS increased phosphorylation of IκB-α and p65 in the cells. As expected, treatment with LUT decreased the LPS-induced phosphorylation of IκB-α and p65.

Moreover, to evaluate the effect of luteolin on the production of pro-inflammatory cytokines in EA.hy926 cells, we tested the level of TNF-α and IL-8 in cell supernatants using ELISA kits. As presented in Fig. 5a, the TNF-α and IL-8 secreted from EA.hy926 cells in LPS group were apparently more abundant than that in the control group (*P*< 0.01). As expected, 5 μM, 10 μM, and 20 μM LUT markedly diminished LPS-stimulated TNF-α and IL-8 level in cell supernatants (*P*< 0.01).

Taken together, LPS could increase TLR4 expression in EA.hy926 cells and promote the activation of its downstream signaling pathways. Our data suggest that luteolin decreased the expression of adhesion molecules and pro-inflammatory cytokines may through inhibiting LPS/TLR4/NF-κB signaling pathway.

## Discussion

Microcirculation plays a crucial role in supplying oxygen and nutrients by blood flow to the tissues to meet their metabolic demands. Many studies have found that microcirculation disturbance is one of the leading causes of multiple diseases. Luteolin (3′, 4′, 5, 7-tetrahydroxyflavone), a kind of flavonoid found in nature, is enriching in many natural plants and widely presented in certain food items, especially in olive, honey and broccoli, providing an essential link between diet and prevention of chronic disease [18]. Previous studies have reported that luteolin may be a vascular protective agent by directly acting on ECs, and it prevents vascular dysfunction likely associated with antioxidant and anti-inflammatory mechanisms [31], and has the vasorelaxant effect through NO production [32]. In our preliminary experiment, we found that luteolin suppressed hypertensive vascular remodeling to protect blood vessels and inhibited TNF-α-induced vascular endothelial inflammation, suggesting luteolin may have a potential effect for improving microcirculatory disturbance.

LPS shredded from Gram-negative bacteria is a factor for microcirculation disturbance, which started with leukocyte recruitment in the microvessels. Leukocyte recruitment is controlled by adhesion molecules on leukocytes and ECs [27]. LPS could activate and up-regulate the expression of adhesion molecules from several families, resulting in rolling and adhesion of leukocytes to ECs [33]. Firstly, leukocytes rolling along the vessel wall is caused by the activation of endothelial and leukocytic selectins, whereas subsequent activation of ICAM-1 and VCAM-1 on ECs and CD11b, CD18 on leukocytes leads to the firm adhesion of leukocytes on the endothelial surface [34, 35]. In addition, the release of pro-inflammatory cytokines such as TNF-α, IL-6 and IL-8 could be stimulated by LPS [27, 36], which further exaggerates the adhesion between leukocytes and ECs followed by leukocytes extravasation and inflammatory reaction. The release of ROS [37] and mast cell degranulation [35] are promoted by the interactions between leukocytes and ECs, which exacerbates microcirculatory disturbance. Accordingly, inhibiting the adhesion between leukocytes and ECs is regarded to be the key to alleviating the disturbance of microcirculation induced by LPS.

Injection of LPS on animals is widely used as a model in the field of microcirculation disturbance research. With this model, the present experiment studied the effects of luteolin on the microcirculatory disturbance in vivo by examining the venular diameter, RBC velocity, leukocyte rolling, leukocyte adhesion, hydrogen peroxide production, mast cell degranulation, intestinal microcirculation blood flow and so on. Additionally, to identify the molecular mechanisms, the expression of adhesion molecules and pro-inflammatory cytokines were studied in an in vitro experiment.

The results from the present study demonstrated that luteolin could significantly inhibit leukocyte adhesion to the venular wall in LPS-induced rats although has no significant effect on venular diameter, RBC velocity and leukocytes rolling. Meanwhile, luteolin could also suppress the adhesion of monocytes to human EA.hy926 ECs in vitro induced by LPS, implying an direct effect of luteolin on inhibiting the leukocytes adhesion to the endothelium. As mentioned above, leukocytes adhesion to endothelium is the key step to inducing microcirculatory disturbance by LPS stimulation. This process is controlled by adhesion molecules on ECs and leukocytes. Furthermore, pro-inflammatory cytokines could exaggerate leukocyte adhesion.

Our results showed that luteolin attenuates the LPS-induced enhancement of adhesion molecules ICAM-1 and VCAM-1 expression in EA.hy926 cells, which was accordant with the reported effect of luteolin in inhibiting the expression of ICAM-1 and VCAM-1 in TNF-α-induced EA.hy926 cells [26]. In addition, some researchers found that luteolin significantly inhibited the expression of VCAM-1 and ICAM-1 in intestinal epithelial cells [38] or pulmonary microvascular ECs [39]. Moreover, luteolin markedly diminished LPS-stimulated TNF-α and IL-8 levels in EA.hy926 cell supernatants. In conclusion, luteolin protects against LPS-induced adhesion between leukocytes and ECs, in both in vitro and in vivo models. This effect of luteolin may be mediated by inhibiting the expression of adhesion molecules and the secretion of pro-inflammatory cytokines from ECs. However, the effect of luteolin on other adhesion molecules and pro-inflammatory cytokines remains to be explored.

It is widely known that LPS-enhanced adhesion molecules expression and pro-inflammatory cytokines secretion are primarily mediated by TLR4/NF-κB signaling pathway [13]. TLR4 is not only an important member of the TLR family but also a receptor for LPS. Myd88 is an indispensable component of the signaling cascade of TLR4 [40]. Activation of TLR4 can trigger downstream Myd88-dependent signaling pathway [41]. The final event is the activation of NF-κB, then the activated NF-κB protein transfers from cytoplasm to nucleus and binds to DNA reaction elements in gene promoter region to promote the gene transcription [42]. The phosphorylation of IκB typically gets involved in the activation of NF-κB. IκB, an inhibitor of NF-κB, is known to mainly bind to P65/P50 heterodimer in the cytoplasm [43, 44]. It is the phosphorylation of IκB that results in its ubiquitylation and subsequent degradation, which allows the release of NF-κB and its translocation to nucleus. Eventually, activation of the NF-κB leads to the production of cell adhesion molecules, such as ICAM-1 and VCAM-1 [45], and secretion of pro-inflammatory cytokines [27, 46], such as TNF-α and IL-8. These cytokines will then cause adhesion between leukocytes and ECs.

Western blot was used to analyze the expression of molecules in TLR4/NF-κB signaling pathway. Our results showed that LPS increased TLR4 expression in EA.hy926 cells and promoted the activation of its downstream signaling pathways. Luteolin could inhibit the expression of TLR4 and Myd88, which consequently suppressed activation of IκB and NF-κB. To further investigate the effect of luteolin on activation of IκB and NF-κB, IκB-α and NF-κB/p65 protein and their phosphorylated form were examined after LPS stimulation. We found that luteolin obviously inhibited LPS-induced phosphorylation and degradation of IκB-α and the activation of NF-κB/p65. In addition, it was reported that luteolin could improve TNF-α-induced vascular inflammation by inhibiting the NF-κB-mediated pathway [26]. Some researchers found that luteolin attenuated the activation of NF-κB and AP-1 in LPS-induced RAW 264.7 cells [47], and ameliorated LPS-mediated nephrotoxicity via decreasing NF-κB activation [48]. These indicated that luteolin could decrease the expression of adhesion molecules and the secretion of pro-inflammatory cytokines by inhibiting TLR4/NF-κB signaling pathway.

The interaction between leukocytes and ECs promotes ROS production [49]. ROS, markedly by the treatment of lute including hydrogen peroxide, causes cytotoxicity in ECs and induces pro-inflammatory cytokine production, which subsequently exacerbates microcirculatory disturbance. DHR fluorescence intensity was detected to quantify the effect of luteolin on hydrogen peroxide production in venular wall. The results showed that luteolin markedly inhibited LPS-induced hydrogen peroxide production on venular wall. Previous studies have reported that luteolin could protect against H_2_O_2_-induced oxidative stress and ameliorated ROS and superoxide generation in HUVECs, and prevent endothelial dysfunction induced by oxidative stress [50]. Therefore, the results of this study indicate that the improvement of luteolin on LPS-induced microcirculatory disorders is not only by inhibition of adhesion between leukocytes and ECs but also by scavenging ROS.

It is acknowledged that LPS makes mast cells degranulate, which leads to the release of cytokines such as histamine and TNF-α. These cytokines launch an extravascular attack on microvessels, promote adhesion molecules expression on ECs, and subsequently enhance leukocytes adhesion and venular hyperpermeability. In the present study, luteolin markedly decreased the percent of LPS-induced degranulated mast cells, suggesting that luteolin can protect against the microcirculatory disturbance by blockage of vasoactive substances from outside. Moreover, luteolin could improve the reduction of intestinal microcirculation blood flow, and it may be associated with the inhibition of leukocyte adhesion. The inhibition of leukocyte adhesion may enlarge the vascular lumen and increase the blood flow. Luteolin also could prevent the inflammatory infiltrates in the mesenteric artery, small intestine edema and fluff disintegration. These pathological changes are all involved in microcirculatory disturbance.

To sum up, this study used the rat inflammation model induced by LPS to evaluate the effect of luteolin on microcirculation disturbance. The results revealed that luteolin could significantly ameliorate microcirculatory disturbance. Interestingly, we found that luteolin could significantly inhibit the leukocyte adhesion to the venular wall, but it had no significant effect on leukocyte rolling. The results of further cell adhesion experiments in vitro were consistent with those in vivo. Thus, we focused on the modulation of the adhesion between leukocytes and ECs and its possible mechanism. As well accepted, LPS-enhanced adhesion molecules expression and pro-inflammatory cytokines secretion, which leads to the firm adhesion of leukocytes to the endothelial surface, are primarily mediated by TLR4/NF-κB signaling pathway. Our results further showed that luteolin could inhibit the expression of TLR4 and Myd88, phosphorylation of IκB-α and NF-κB/p65, and the expression of ICAM-1, VCAM-1, TNF-α and IL-8, suggesting luteolin could decrease the expression of adhesion molecules and pro-inflammatory cytokines by inhibiting LPS/TLR4/NF-κB signaling pathway. These results are consistent with the anti-inflammatory effects of luteolin previously reported.

**Fig. 6:**
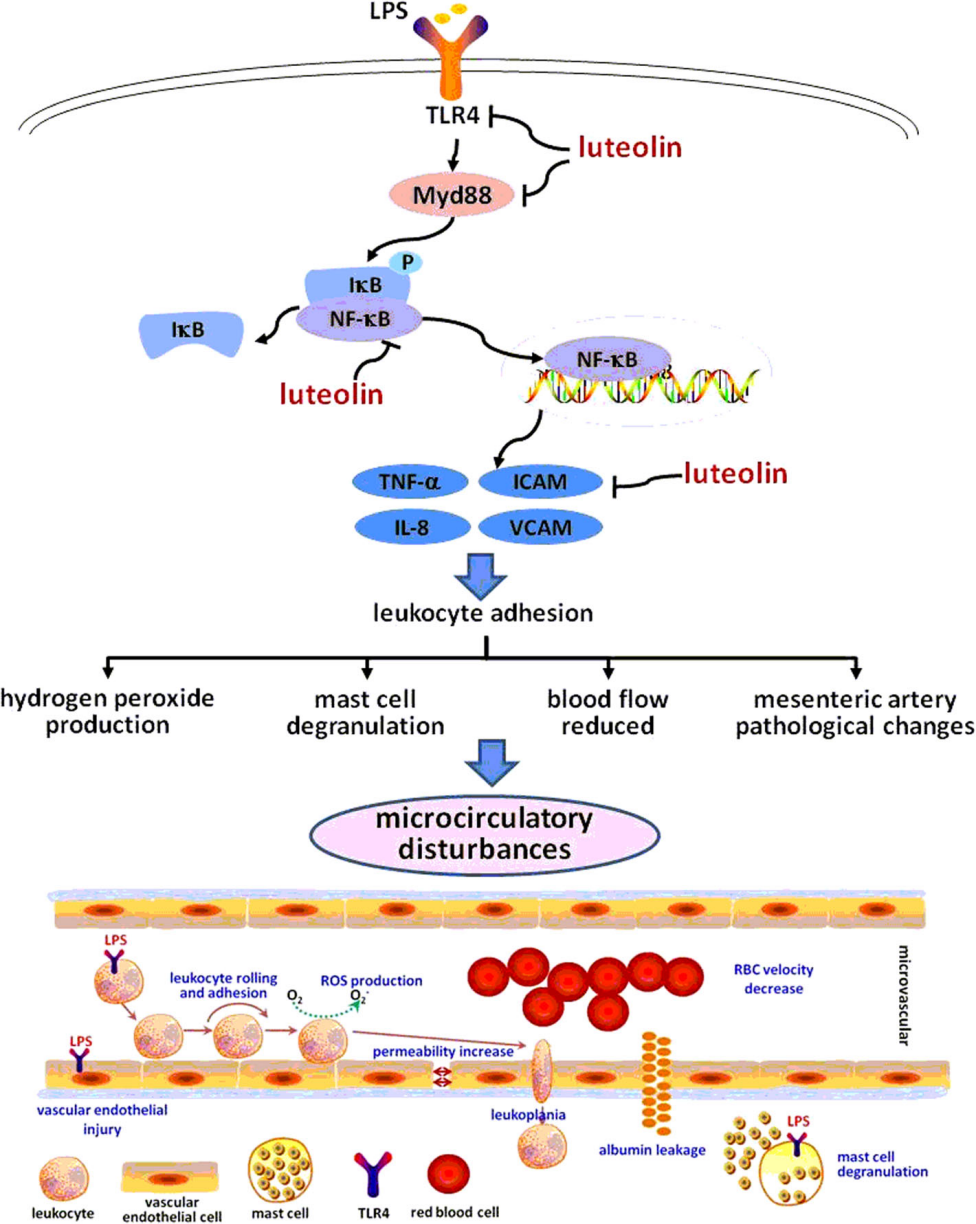
The proposed mechanism of luteolin on suppressing microcirculatory disturbance caused by cell adhesion. Luteolin could decrease the expression of adhesion molecules and pro-inflammatory cytokines by inhibiting LPS/TLR4/NF-κB signaling pathway, which would suppress LPS-induced leukocyte adhesion. Subsequently, the other processes of microcirculatory disturbance induced by LPS such as hydrogen peroxide production, mast cell degranulation, blood flow reduced and mesenteric artery pathological changes were also improved

Microcirculatory disturbance induced by LPS is a complex pathological process, which may initiates with leukocyte-EC interaction, followed by reactive oxygen species production, mast cell degranulation, microvessel hyperpermeability, albumin extravasation and so on. These processes could interact. However, leukocytes adhesion to endothelial cells is a crucial contribution to the development of microcirculatory disturbance. Based on the results of our study, we speculated luteolin improved LPS-induced microcirculation disturbance possibly by inhibiting leukocyte adhesion through the regulation of LPS/TLR4/NF-κB signaling pathway, as shown in Fig.6. However, due to existing deficiencies such as limited methods and data, more researches are needed to corroborate this mechanism of luteolin, and other mechanisms remain to be explored.

## Conclusions

Our studies concluded that luteolin inhibited the LPS-induced activation of the TLR4/Myd88/NF-κB signaling pathway. It down-regulated the expression of TLR4, Myd88 and the phosphorylation of IκB-a and NF-κB, inhibited the expression of cell adhesion molecules including ICAM-1 and VCAM-1, and restrained pro-inflammatory cytokine production including TNF-α and IL-8. Furthermore, luteolin suppressed LPS-induced adhesion between leukocytes and ECs, as well as hydrogen peroxide production. In addition, luteolin inhibited the venular hyperpermeability, decreased mast cell degranulation, increased intestinal microcirculation blood flow, and improved mesenteric artery and small intestine pathological changes. Finally, luteolin ameliorated microcirculatory disturbance. The findings in our studies indicate that luteolin is a potential inhibitor of cell adhesion, and may have potential application in treating microcirculatory disturbance.

## Supplementary Information

**Additional file 1.**

## Data Availability

The data used and/or investigated during the present study are accessible from the corresponding author on reasonable request.

## Abbreviations

LPS: Lipopolysaccharide
TLR4: Toll-like receptor 4
ICAM-1: Intercellular adhesion molecule 1
VCAM-1: Vascular cell adhesion molecule 1
TNF-α: Tumor necrosis factor-alpha
NF-κB: Nuclear factor-kappa B
MyD88: Myeloid differentiation factor 88
IκB: Inhibitor of Nuclear factor-kappa B
IL-8: Interleukin-8
LUT: Luteolin
TLUT: Luteolin enriched extracts
DHR: Dihydrorhodamine 123
RBC: Red blood cell
EA.hy926: Human umbilical vein cell fusion cell
THP-1: Human monocytic leukemia cell
