# Supplementary material for: Luteolin ameliorates lipopolysaccharide-induced microcirculatory disturbance through inhibiting leukocyte adhesion in rat mesenteric venules
Source: BMC Complement Med Ther. 2021 Jan 14;21:33. doi: 10.1186/s12906-020-03196-9 (PMC7807763; doi:10.1186/s12906-020-03196-9)
Supplement: Supplementary file 1 — Additional file 1. [file 12906_2020_3196_MOESM1_ESM.docx]

Supplementary Fig.7 Original Western blots used for Fig.5


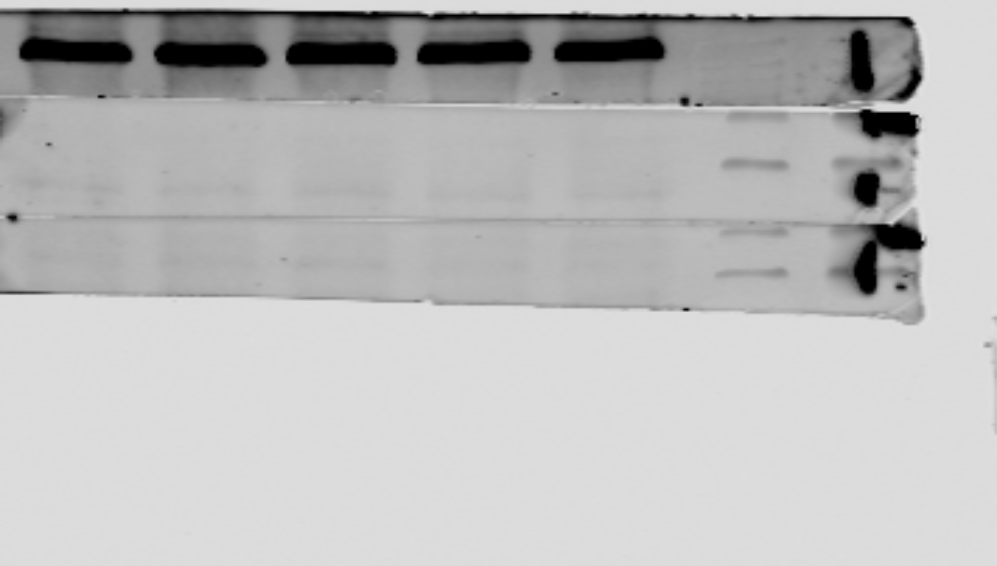


LUT 20

control

LPS

LUT 5

LUT 10

Fig.7A β-actin original blot image

Samples are labelled throughout as follows.

control: control group

LPS: LPS 3 μg/mL group

LUT 5: LPS 3 μg/mL + LUT 5 μM group

LUT 10: LPS 3 μg/mL + LUT 10 μM group

LUT 20: LPS 3 μg/mL + LUT 20 μM group

All proteins are the same batch of samples. The protein as β-actin bands marked by red line are used for this manuscript.


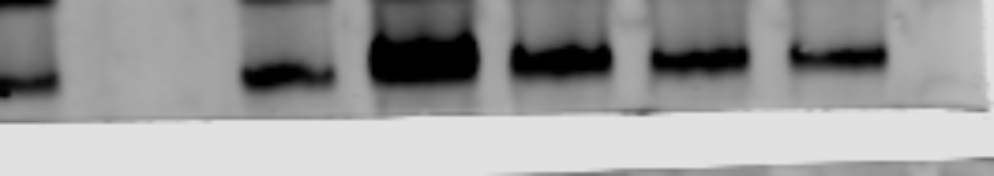


LUT 20

LUT 10

LUT 5

LPS

control

Fig.7B TLR4 original blot image

Samples are labelled throughout as follows.

control: control group

LPS: LPS 3 μg/mL group

LUT 5: LPS 3 μg/mL + LUT 5 μM group

LUT 10: LPS 3 μg/mL + LUT 10 μM group

LUT 20: LPS 3 μg/mL + LUT 20 μM group

All proteins are the same batch of samples. The protein as TLR4 bands marked by red line are used for this manuscript.


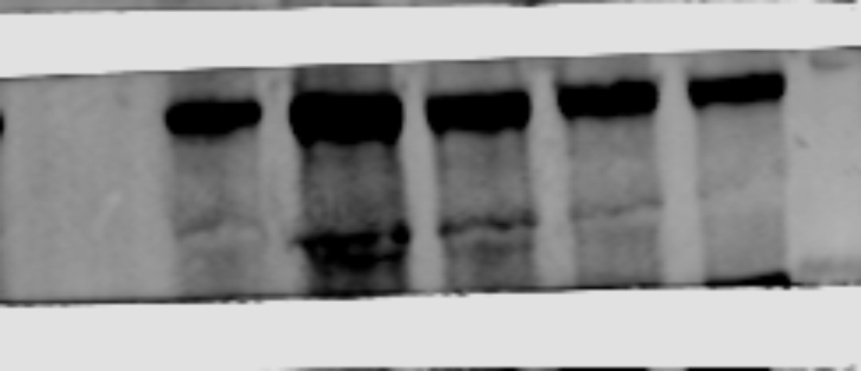


control

LPS

LUT 20

LUT 10

LUT 5

Fig.7C Myd88 original blot image

Samples are labelled throughout as follows.

control: control group

LPS: LPS 3 μg/mL group

LUT 5: LPS 3 μg/mL + LUT 5 μM group

LUT 10: LPS 3 μg/mL + LUT 10 μM group

LUT 20: LPS 3 μg/mL + LUT 20 μM group

All proteins are the same batch of samples. The protein as Myd88 bands marked by red line are used for this manuscript.


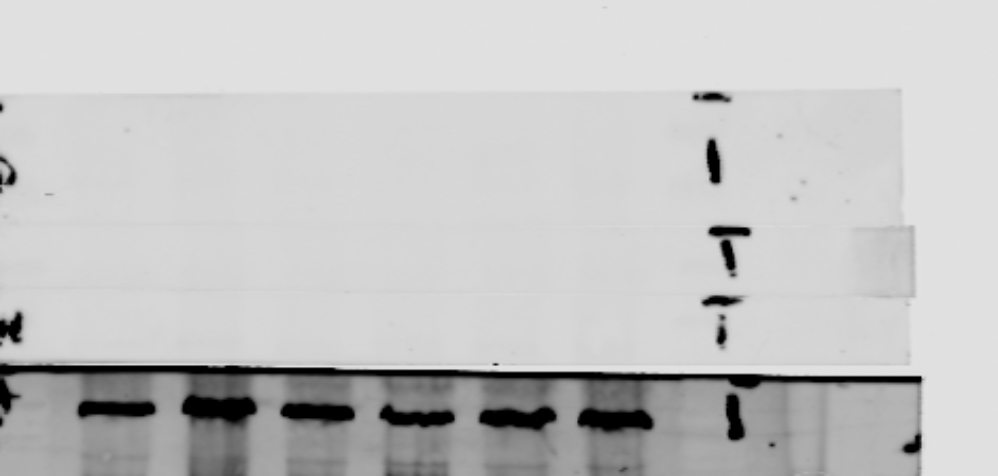


LPS

LUT 10

LUT 20

LUT 5

LPS

control

Fig.7D p65 original blot image

Samples are labelled throughout as follows.

control: control group

LPS: LPS 3 μg/mL group

LUT 5: LPS 3 μg/mL + LUT 5 μM group

LUT 10: LPS 3 μg/mL + LUT 10 μM group

LUT 20: LPS 3 μg/mL + LUT 20 μM group

All proteins are the same batch of samples. The protein as p65 bands marked by red line are used for this manuscript. The other band is the duplicate sample as LPS group, and not used for this manuscript.


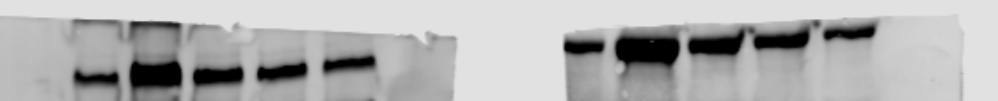


LUT 5

LUT 20

LUT 10

LPS

control

Fig.7E ICAM-1 original blot image

Samples are labelled throughout as follows.

control: control group

LPS: LPS 3 μg/mL group

LUT 5: LPS 3 μg/mL + LUT 5 μM group

LUT 10: LPS 3 μg/mL + LUT 10 μM group

LUT 20: LPS 3 μg/mL + LUT 20 μM group

All proteins are the same batch of samples. The protein as ICAM-1 bands marked by red line are used for this manuscript. The other bands are other proteins, not related to this manuscript.


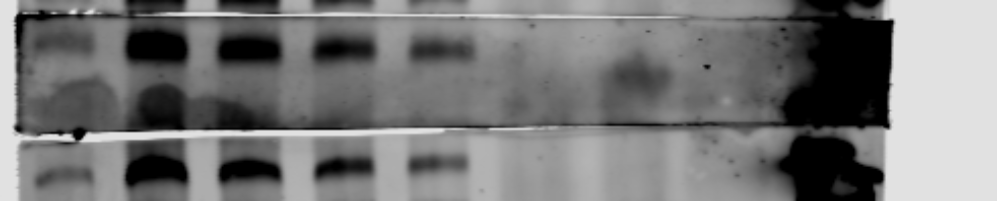


LUT 20

LUT 10

control

LUT 5

LPS

Fig.7F p-IκB-α original blot image

Samples are labelled throughout as follows.

control: control group

LPS: LPS 3 μg/mL group

LUT 5: LPS 3 μg/mL + LUT 5 μM group

LUT 10: LPS 3 μg/mL + LUT 10 μM group

LUT 20: LPS 3 μg/mL + LUT 20 μM group

All proteins are the same batch of samples. The protein as p-IκB-α bands marked by red line are used for this manuscript. The other bands are other proteins, not related to this manuscript.


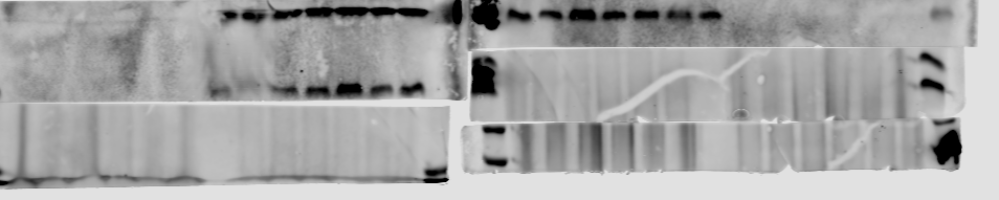


LPS

LPS

LPS

control

LUT

20

LUT

5

LUT

10

Fig.7G IκB-α original blot image

Samples are labelled throughout as follows.

control: control group

LPS: LPS 3 μg/mL group

LUT 5: LPS 3 μg/mL + LUT 5 μM group

LUT 10: LPS 3 μg/mL + LUT 10 μM group

LUT 20: LPS 3 μg/mL + LUT 20 μM group

All proteins are the same batch of samples. The protein as IκB-α bands marked by red line are used for this manuscript. Two bands are the duplicate samples as LPS group, and the other bands are other proteins, not related to this manuscript.


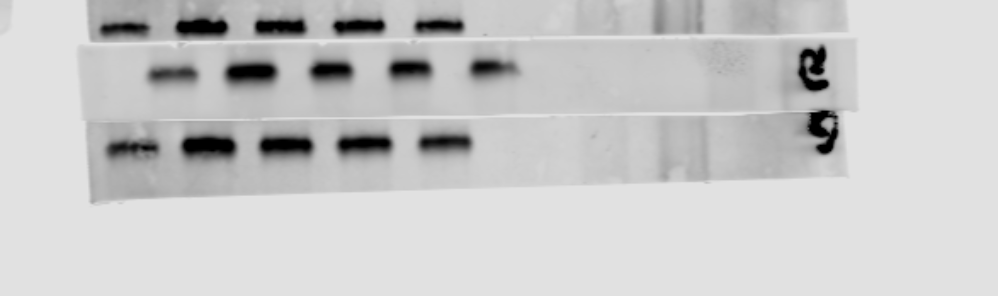


LUT 20

LUT 10

LUT 5

LPS

control

LUT 20

LUT 10

LUT 5

LPS

control

Fig.7H VCAM-1 and p-p65 original blot image

Samples are labelled throughout as follows.

control: control group

LPS: LPS 3 μg/mL group

LUT 5: LPS 3 μg/mL + LUT 5 μM group

LUT 10: LPS 3 μg/mL + LUT 10 μM group

LUT 20: LPS 3 μg/mL + LUT 20 μM group

All proteins are the same batch of samples. The protein as VCAM-1 bands marked by red line and the protein as p-p65 bands marked by blue line are used for this manuscript. The other bands are other proteins, not related to this manuscript.

Additional images

The original photographs using the LI-COR Odyssey CLx Infrared Imaging System


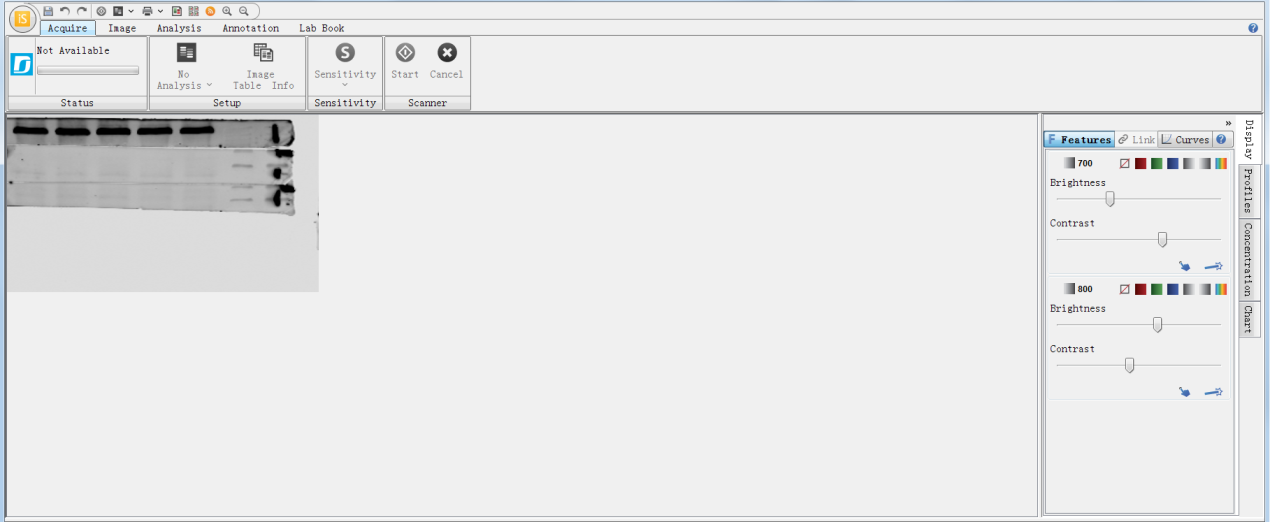


β-actin


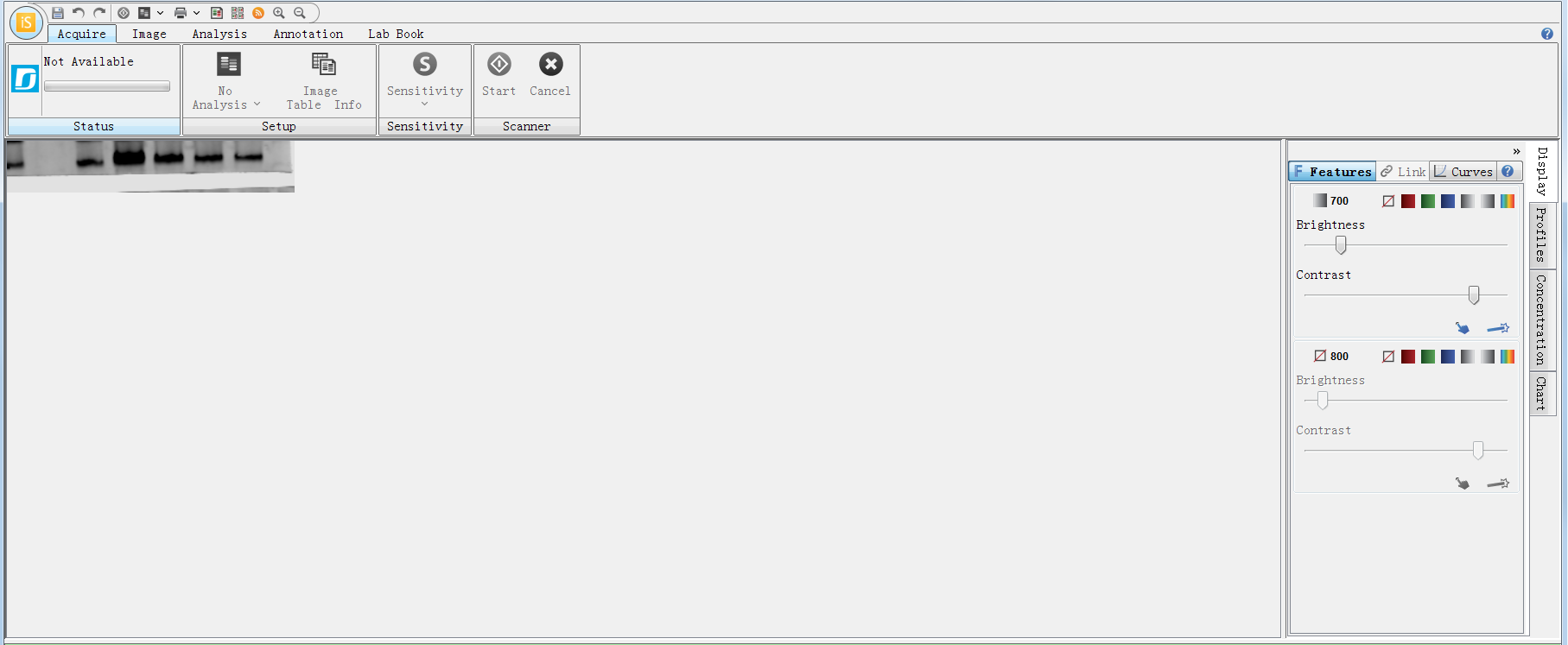


TLR4


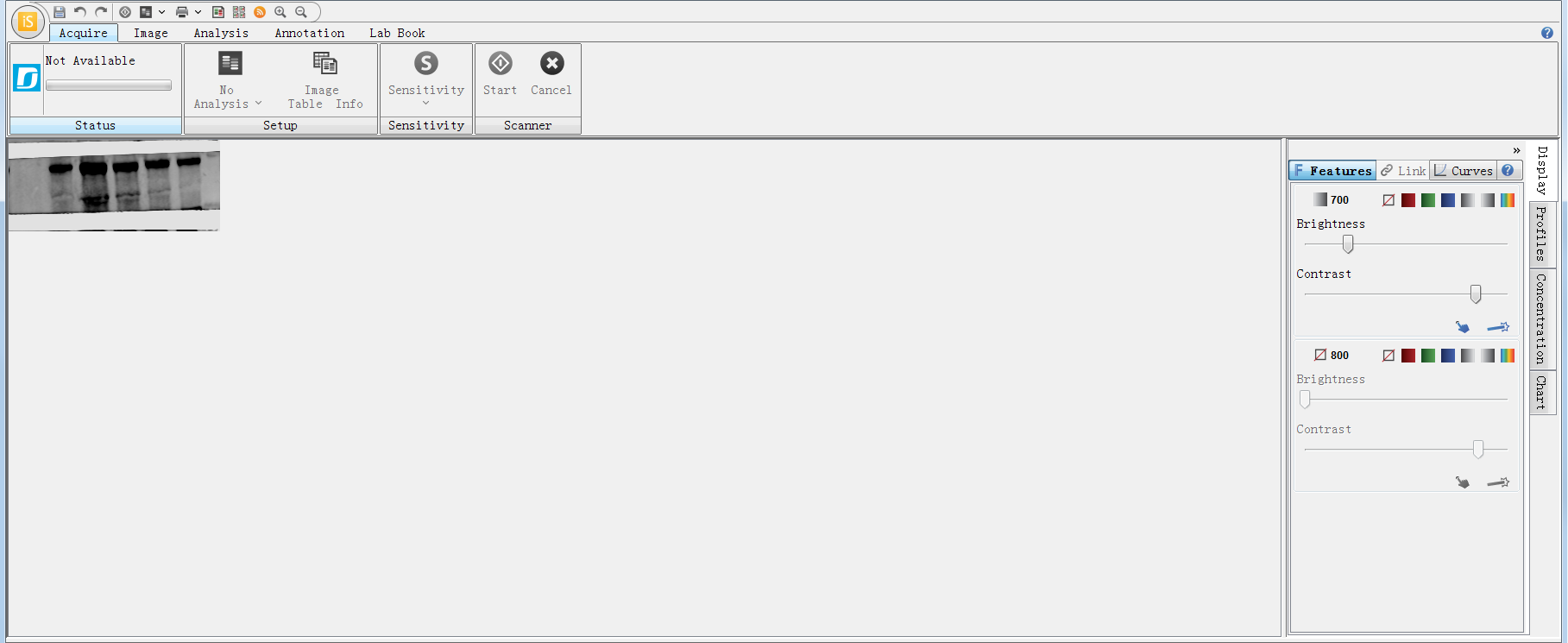


Myd88


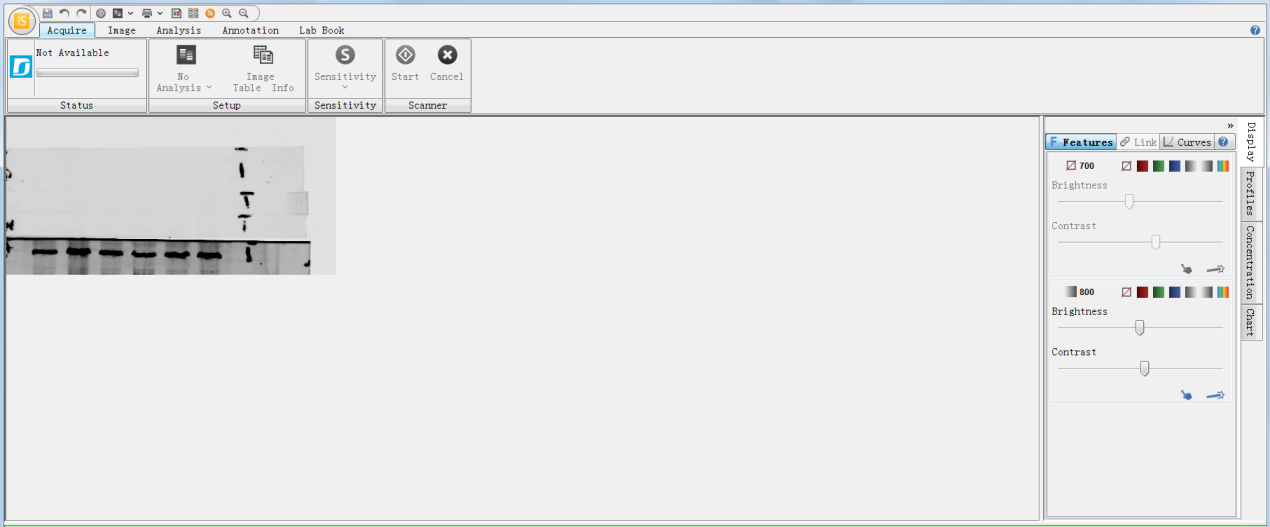


p65


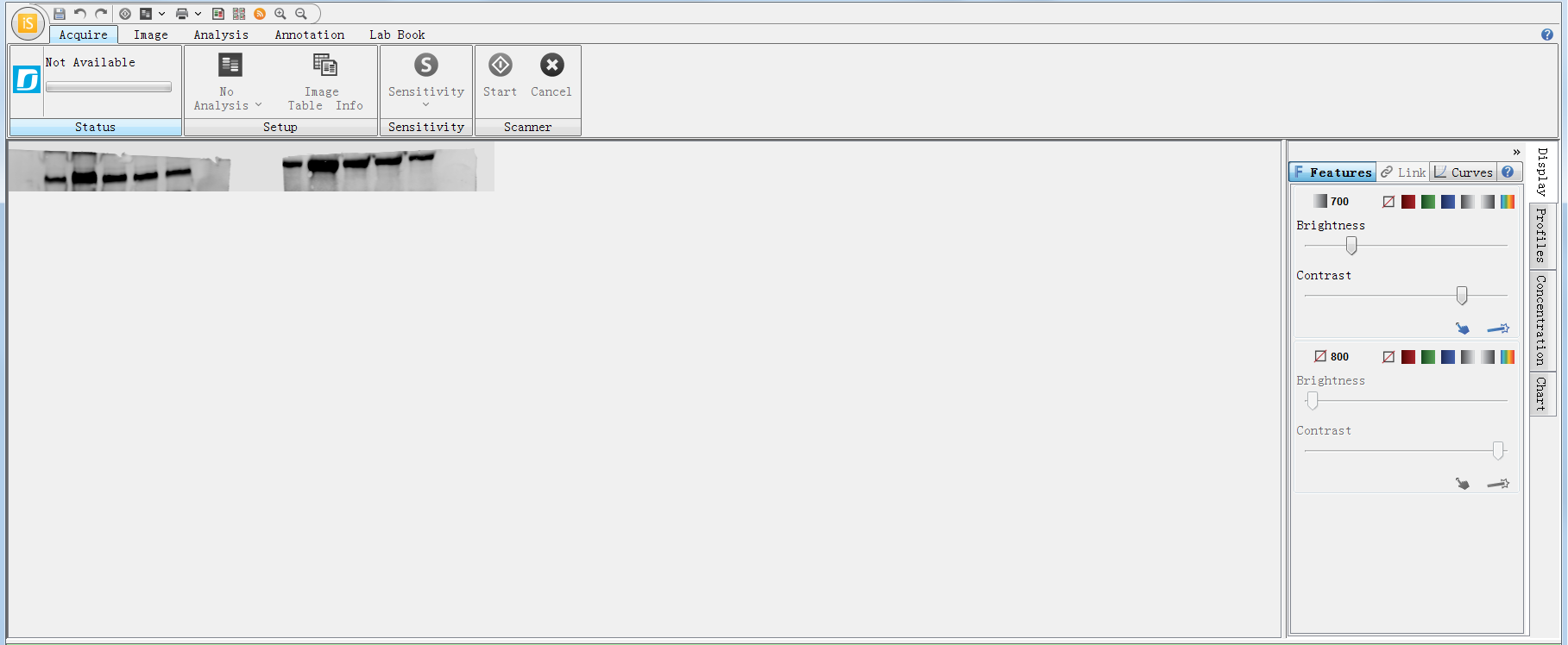


ICAM-1


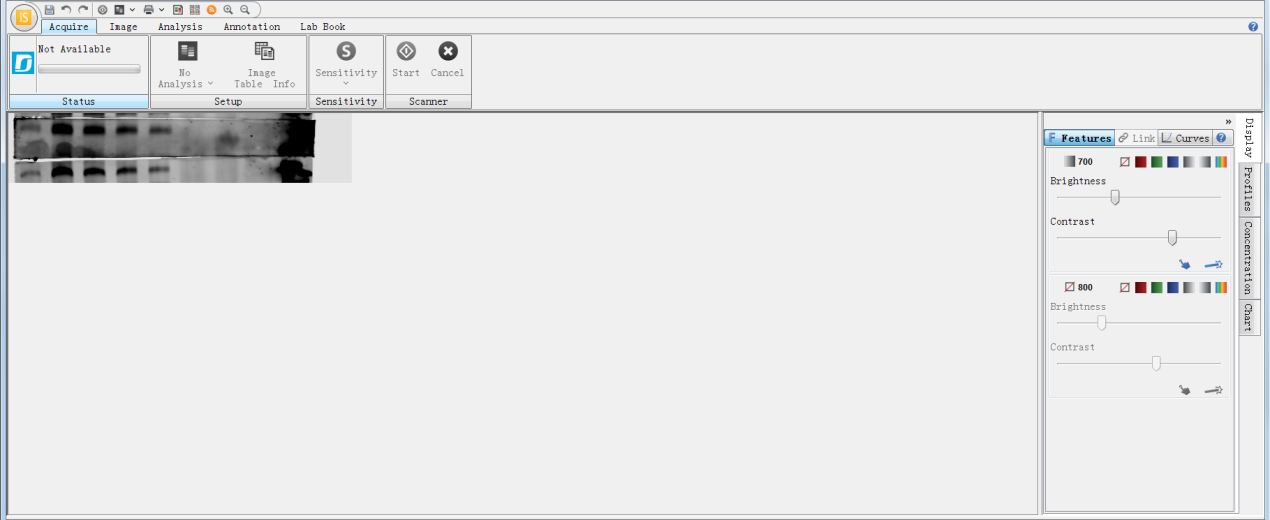


p-IκB-α


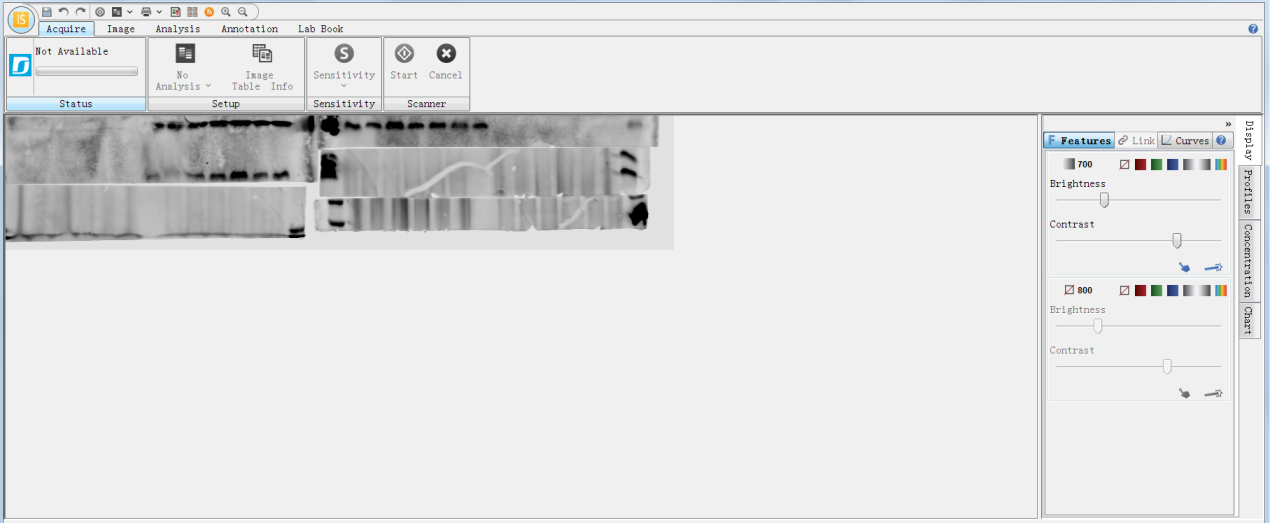


IκB-α


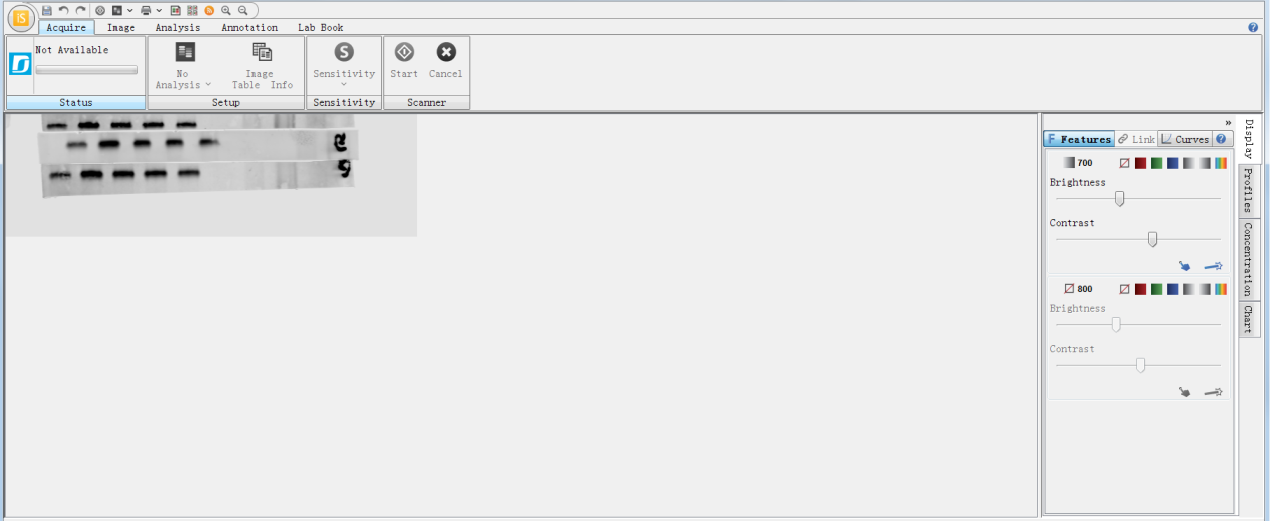


VCAM-1 and p-p65
